# PHARMACIST INTERVENTION IS ASSOCIATED WITH MEDICATION ADHERENCE IN OLDER ADULTS WITH CHRONIC CONDITIONS

**DOI:** 10.1093/geroni/igad104.3523

**Published:** 2023-12-21

**Authors:** Renae Smith-ray, Liang Feng, Tanya Singh, Kristi Rudkin, Stacey Emmons, Erik Groves, Heather Kirkham

**Affiliations:** Walgreens, Deerfield, Illinois, United States; Walgreens, Deerfield, Illinois, United States; Walgreens, Deerfield, Illinois, United States; Walgreens, Deerfield, Illinois, United States; Walgreens, Deerfield, Illinois, United States; Walgreens, Deerfield, Illinois, United States; Walgreens, Deerfield, Illinois, United States

## Abstract

Hypertension, hyperlipidemia, and type 2 diabetes (T2D) are prevalent chronic diseases among older Americans, but related medication adherence rates are less than optimal. Consequences of poor adherence include increased mortality, healthcare utilization, and costs. Compounding evidence supports the integration of pharmacists in patients’ care across the care continuum. This study’s purpose was to examine the impact of a large national pharmacy chain’s pharmacist-led interventions to improve medication adherence among older adults with hypertension, hyperlipidemia, or T2D. Participants were Medicare D patients who had ≥2 prescription fills in ≥1 of the three therapeutic classes. The primary outcome, optimal adherence (OA), was defined as proportion of days covered (PDC) ≥80%. A difference-in-differences (DID) design with a generalized linear model approach was applied to examine differences between participants exposed to the intervention and controls across the study period (2020-2022). At baseline, intervention participants (n=317,613, age 70.1, female 57.0%) had lower OA for diabetes (76.9% vs. 79.8%), hypertension (79.0% vs. 83.0%), and cholesterol (78.6% vs. 82.1%) compared to controls (n=943.389, age 73.3, female 56.1%). The DID results showed that between 2020 - 2022, OA had significant absolute increases for intervention participants (diabetes: +4.0%, hypertension: +6.3%, cholesterol: +6.1%) versus controls (diabetes: -1.6%, hypertension: -0.4%, cholesterol: -1.4%). All DID models were significant at p< 0.0001. The pharmacist-led intervention was significantly associated with increased OA over 2 years. These findings reinforce the potential for pharmacists as care providers among older adults with chronic conditions. Future research should examine the expansion of pharmacist-provided care.

